# Optical Coherence Tomography in Parkinsonian Syndromes

**DOI:** 10.1371/journal.pone.0034891

**Published:** 2012-04-13

**Authors:** Philipp Albrecht, Ann-Kristin Müller, Martin Südmeyer, Stefano Ferrea, Marius Ringelstein, Eva Cohn, Orhan Aktas, Thomas Dietlein, Alexandra Lappas, Andreas Foerster, Hans-Peter Hartung, Alfons Schnitzler, Axel Methner

**Affiliations:** 1 Department of Neurology, Medical Faculty, Heinrich-Heine University Düsseldorf, Düsseldorf, Germany; 2 Department of Ophthalmology, Medical Faculty, Heinrich-Heine University Düsseldorf, Düsseldorf, Germany; 3 Department of Ophthalmology, University Hospital of Cologne, Cologne, Germany; 4 Institute of Clinical Neuroscience and Medical Psychology, Medical Faculty, Heinrich-Heine University Düsseldorf, Düsseldorf, Germany; Charité University Medicine Berlin, Germany

## Abstract

**Background/Objective:**

Parkinson's disease (PD) and the atypical parkinsonian syndromes multiple system atrophy (MSA), progressive supranuclear palsy (PSP) and corticobasal syndrome (CBS) are movement disorders associated with degeneration of the central nervous system. Degeneration of the retina has not been systematically compared in these diseases.

**Methods:**

This cross-sectional study used spectral-domain optical coherence tomography with manual segmentation to measure the peripapillar nerve fiber layer, the macular thickness, and the thickness of all retinal layers in foveal scans of 40 patients with PD, 19 with MSA, 10 with CBS, 15 with PSP, and 35 age- and sex-matched controls.

**Results:**

The mean paramacular thickness and volume were reduced in PSP while the mean RNFL did not differ significantly between groups. In PSP patients, the complex of retinal ganglion cell- and inner plexiform layer and the outer nuclear layer was reduced. In PD, the inner nuclear layer was thicker than in controls, MSA and PSP. Using the ratio between the outer nuclear layer and the outer plexiform layer with a cut-off at 3.1 and the additional constraint that the inner nuclear layer be under 46 µm, we were able to differentiate PSP from PD in our patient sample with a sensitivity of 96% and a specificity of 70%.

**Conclusion:**

Different parkinsonian syndromes are associated with distinct changes in retinal morphology. These findings may serve to facilitate the differential diagnosis of parkinsonian syndromes and give insight into the degenerative processes of patients with atypical parkinsonian syndromes.

## Introduction

Parkinson's disease (PD) and the atypical parkinsonian syndromes multiple system atrophy (MSA), progressive supranuclear palsy (PSP) and corticobasal syndrome (CBS) are movement disorders associated with degeneration of the central nervous system. Several volumetric MRI studies have reported differences between the different parkinsonian syndromes [1], [2], [3], [4]. In all four diseases, abnormalities of the visual system have been described. In PD, prolonged latencies of visually evoked potentials (VEP) [5] and deficits in contrast sensitivity and color vision [6], [7] have been reported. Also, the function of retinal ganglion cells seems to be altered as shown by pattern electroretinograms (ERG) [8], [9], [10], [11]. In a patient with MSA, optic disc pallor and negative ERGs were observed [12]. Visual event related potentials using a visual oddball paradigm were reported to be abnormal in all four diseases [13]. The often-observed blurry vision of PSP patients is usually attributed to the pronounced impairment of ocular movements and diplopia [14], but might also be associated with retinal changes. PSP and CBS have been reported to be associated with visuospatial deficits [15]. A post-mortem study using high performance liquid chromatography suggested a reduction in the dopamine content of retinas of PD patients [16].

Optical coherence tomography (OCT) is a non-invasive optical interferometric method generating cross-sectional images of the retina in vivo, which has revolutionized retinal imaging since its introduction in the 1990s [17]. Since then OCT has been applied to a variety of disorders of the central nervous system (CNS) with an emphasis on chronic inflammatory CNS diseases like multiple sclerosis (MS) [18], [19], [20], [21], [22], [23]. Several small studies examined the retinal morphology in PD using OCT with different results: Two studies found a significant reduction of the peripapillar retinal nerve fiber layer (RNFL) thickness in 10 and 16 patients with PD only in the inferior and temporal parts of the retina [24], [25]. Others reported a reduction of the mean total peripapillar RNFL thickness and macular volume in 17 PD patients and an inverse correlation of macular thickness with part III of the unified Parkinson's disease rating scale (UPDRS-III) [26]. Another study with 24 PD patients reported thinning of the parafoveal inner retinal layers consisting of the RNFL, the retinal ganglion cell layer (RGC), and the inner plexiform layer (IPL) using Fourier domain OCT [27]. However, others could not reproduce these changes [28], [29]. Recently, a small OCT study with 10 patients reported a reduction of the RNFL thickness in MSA [30]. In our study, we used last-generation spectral-domain OCT to analyze the largest collective of PD patients to date and included patients suffering from atypical parkinsonian syndromes including MSA, PSP, and CBS.

We present a complete dataset of the retinal parameters routinely accessible to modern OCT technology and additionally manually measured the thickness of the different retinal layers in cross-sectional foveal scans, which demonstrated profound differences between these diseases. The strength of this study is that we analyzed all parafoveal retinal layers and systematically compared typical and atypical parkinsonian syndromes.

## Materials and Methods

### Ethics

Patients examined in this study were recruited from a large longitudinal study on patients with movement disorders, approved by the local ethics committee, the “Ethikkommission der Medizinischen Fakultät der Heinrich-Heine-Universität Düsseldorf, Ethikkommission@med.uni-duesseldorf.de". Within this study, we performed morphometric MRI scans, neuropsychologic tests and at one time point optical coherence tomography (OCT) measurements. OCT is a fast, non-invasive and not painful method and is included in the routine diagnostic program for patients with movement disorders at our center. Written informed consent was given by all subjects for data acquisition, MRI morphometry, and clinical and neuropsychologic examinations. Before the actual OCT examination, the procedure and the reason for this examination in particular were explained to the patients in full detail and additional informed consent was obtained verbally. This procedure was approved by the local ethics committee in an amendment to the initial ethics votum.

### Patients

We examined 40 patients with PD, 19 with MSA, 10 with CBS, 15 with PSP, and 35 control patients without ophthalmologic, inflammatory or degenerative neurological disease after obtaining informed consent. They were consecutively recruited between 2009 and 2011 at the University Hospital Düsseldorf, Germany. All patients were clinically diagnosed and underwent long-term follow-up examinations (mean follow up period 25±2 months) and the final diagnosis was established by the consensus of three movement disorder specialists who were unaware of the OCT results. PD, MSA, and PSP were diagnosed following the established criteria [31], [32], [33], [34]. Subjects with progressive asymmetric parkinsonism, ideomotor apraxia and possible further symptoms like dystonia, alien limb phenomenon, cortical sensory loss, or myoclonia were classified as CBS [35]. All diagnoses were established taking into consideration data of the longitudinal neurologic follow-up examinations, possible response to dopaminergic treatment, diagnostic MRI, and in most cases scintigraphy (FP-CIT-, IBZM- and MIBG-SPECT). Of the patients with MSA, four had the cerebellar (MSA-C) and 16 the parkinsonian (MSA-P) subtype. The UPDRS-III was determined after 12 h discontinuation of anti-parkinsonian therapy [36], levodopa responsiveness by a short-term levodopa test or by a clear qualitative levodopa response during clinical follow-up. Corrected visual acuity was assessed using Snellon charts and was above 6/10 for all eyes. All patients received funduscopy and reported formal ophthalmologic exams within three years before the examination. Subjects with a history of retinopathy or glaucoma or high myopia were excluded from the analysis and the 10% with the lowest values for macular thickness and RNFL of each group underwent a second formal ophthalmologic examination including slit lamp, visus and tonometry after OCT to exclude a confounding ocular pathology.

### Optical coherence tomography

**Figure 1:**
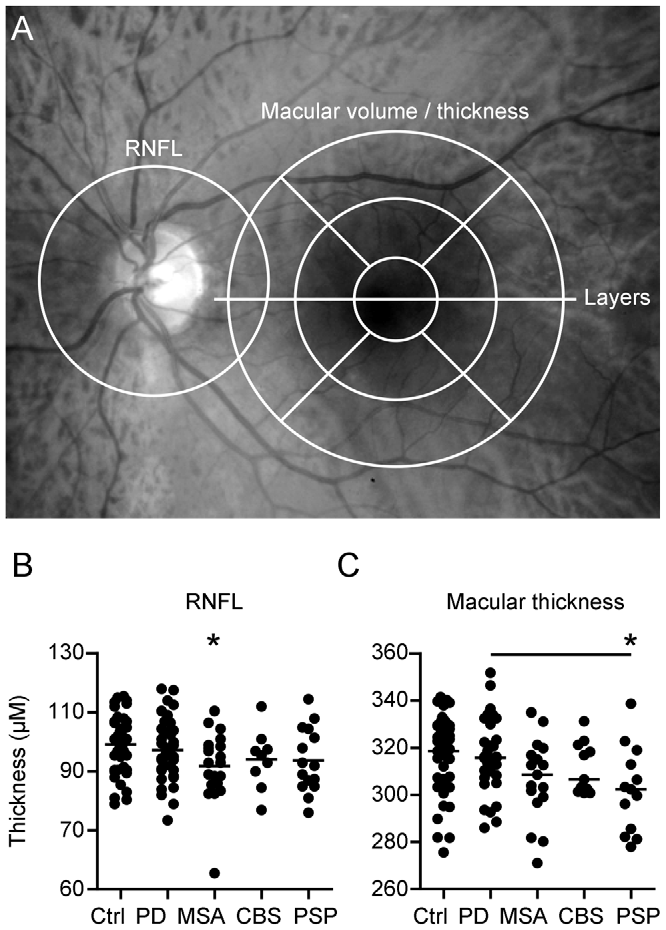
Macular thickness is reduced in PSP. (**A**) The areas of measurement are marked in an image of the fundus: The RNFL was measured in a circular scan centered on the optic disc, macular thickness and volume in scans around the macula, the manual segmentation of the retinal layers was performed in a horizontal scan through the center of the fovea. Scatter plots display the thickness of the RNFL (**B**) and the total macular thickness (**C**). Each point represents the mean of the two eyes of one patient. The mean of all patients is indicated by a horizontal bar. Significant difference to the control group is indicated by asterisks (*p*<0.05, ANOVA and Dunnett's post-hoc test) and significant difference between the patient groups by horizontal bars (*p*<0.05, ANOVA and Tukey's post-hoc test).

The details of the principles of spectral-domain OCT have been described elsewhere [37]. Using a Spectralis OCT device (Heidelberg Engineering, Heidelberg, Germany) with image alignment eye tracking-software (TruTrack, Heidelberg Engineering, Heidelberg, Germany), we obtained perifoveal volumetric retinal scans consisting of 25 single horizontal axial scans (scanning area: 6×6 mm, centered at the fovea, figure 1). To assess the peripapillar RNFL, a circular scan with a diameter of approximately 3.4 mm was performed after manually positioning the center on the middle of the optic disc (figure 1). Furthermore, we performed high-resolution horizontal scans through the middle of the fovea. All scans were performed with support of the eye-tracking system, RNFL measurements and high resolution single horizontal scans were averaged from 100 images and scans for volumetric calculations from 10 (Automatic Real Time, ART). Scans with poor quality (<20 DB) were excluded from the analysis.

**Figure 2:**
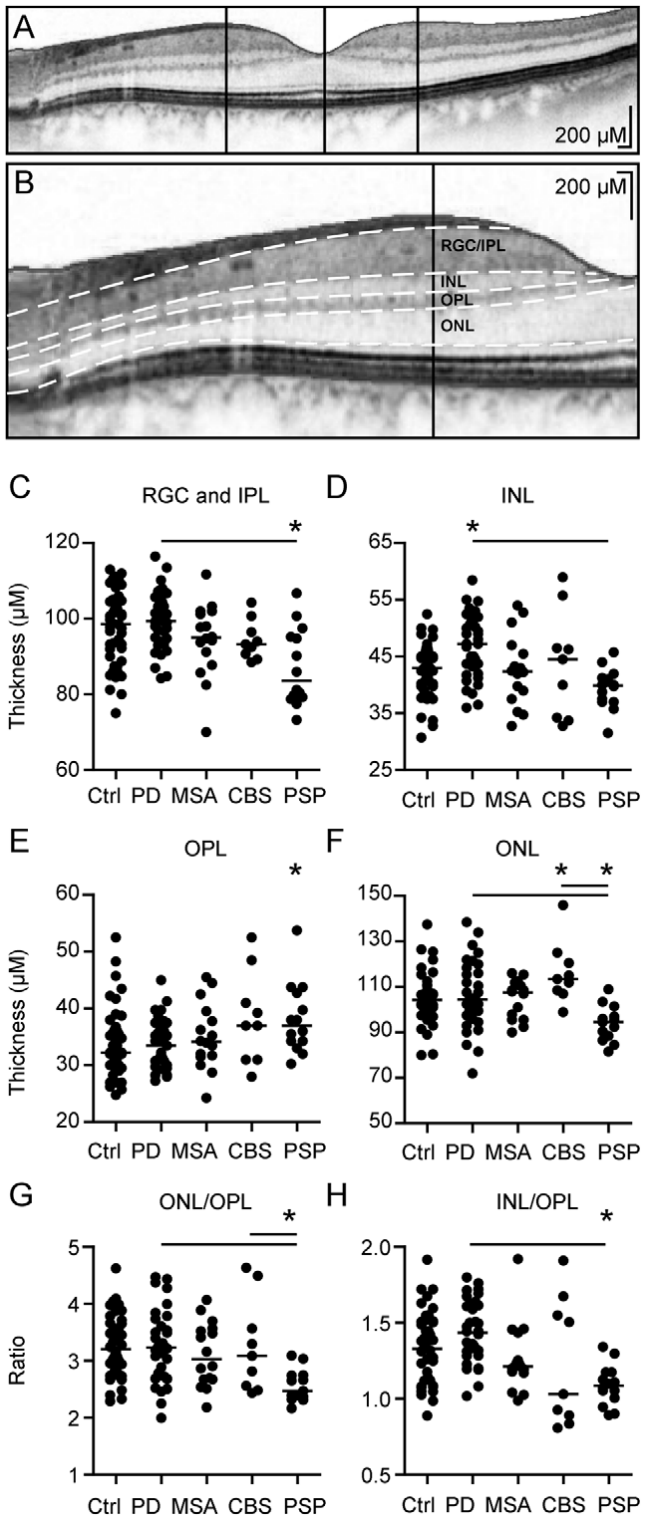
Manual segmentation of the parafoveal retinal layers. **A and B** The segmentation of the different retinal layers was performed manually in single horizontal foveal scans, the images are displayed as negatives to better differentiate the different layers. **B** shows a close-up of **A** after manual segmentation, the retinal layers are divided by white dotted lines. The thickness of the different layers was measured at the vertical lines indicating the thickest point nasally and temporally of the fovea, except for the ONL, which was measured centrally along the vertical line. **C–F** Scatter plots of the mean thickness of the different retinal layers. Each point represents the mean of the two eyes of one patient. The mean of all patients is indicated by a horizontal bar. Significant difference to the control group is indicated by asterisks (*p*<0.05, ANOVA and Dunnett's post-hoc test) and significant difference between the patient groups by horizontal bars (*p*<0.05, ANOVA and Tukey's post-hoc test). **G–H** As some of the retinal layers showed reverse changes in the different Parkinson syndromes. We calculated their ratios to better discriminate the groups. **G** and **H** show scatter plots of the ratio between the mean ONL/OPL thickness and the mean INL/OPL thickness. Each point represents the mean of the two eyes of one patient. The mean of all patients is indicated by a horizontal bar. Significant difference to the control group is indicated by asterisks (*p*<0.05, ANOVA and Dunnett's post-hoc test) and significant difference between the patient groups by horizontal bars (*p*<0.05, ANOVA and Tukey's post-hoc test).

While the results of the RNFL- and paramacular volumetric measurements were automatically segmented, the segmentation of the different retinal layers in the single horizontal foveal scans was performed manually by repositioning the measurement lines (white dotted lines in figure 2) on the borders between the different layers. The thickness of the different layers was measured at the thickest point nasally and temporally of the macula using Heidelberg Eye explorer software (vertical black lines in figure 2). As in most subjects the outer nuclear layer (ONL) presents only one central thickest point rather than nasal and temporal peaks as in the other layers, we used the thickness at this point for analysis (central vertical line in figure 2). In the few subjects with a nasal and a temporal maximum of thickness, we used the higher value.

To assure test-retest reliability of the manual segmentation method, 19 subjects were examined twice and the results for each layer were correlated (Spearman).

### Statistical Evaluation

Statistical analysis was performed using Microsoft Excel and Prism 5.0 (Graphpad). Both eyes of each subject were included into the analysis as statistically dependent duplicates. Analysis of variance (ANOVA) was used with Dunnett's multiple comparison post-hoc test for comparison of all groups to controls and with Tukey's multiple comparison post-hoc test for comparison between all pairs of columns. *p*-values below 0.05 were considered as significant. In case of missing data the subject was excluded from the respective analysis.

Sensitivity and specificity were calculated using the two-by-two table method. The positive likelihood ratio (LR+) was calculated as sensitivity/(1-specificity), the negative likelihood ratio (LR−) as (1-sensitivity)/specificity. LR+ above 50 and LR− below 0.05 were considered excellent, LR+ from 10 to 50 and LR− from 0.05 to 0.1 were considered good, and LR+ between 2 and 10 and LR− between 0.1 and 0.3 fair.

## Results

The patient groups and controls did not differ significantly in age (exact age is depicted in supplemental table S1 and means in table 1). The sex ratio did not differ significantly between controls and/or patient groups except between MSA and CBS. We observed no differences of any retinal layer between men and women in our control group. Patients did not differ in disease severity (UPDRS-Off). Disease duration was longer in PD, but did not differ between the other groups (table 1, supplemental table S1).

**Table 1:** Clinical parameters.

	Age, y	Duration, m	Follow up, m	H/Y scale	UPDRSIII ON	UPDRSIII OFF
PD	61.2+−2.0	8.1+−0.8	30.8+−3.0	2.5+−0.2	18.4+−1.3	35.7+−3.3
MSA	63.2+−1.7	4.3+−0.1	23.2+−3.7	3.2+−0.2	36.4+−3.8	33.4+−4.4
CBS	63.2+−2.4	2.7+−1.5	18.4+−3.7	2.3+−0.3	27.2+−4.5	35.0+−5.7
PSP	71.3+−1.5	4.3+−3.3	16.7+−3.6	2.8+−0.3	29.2+−3.5	36.0+−5.6

The means and the standard error of the means are indicated for the clinical parameters of the different Parkinson syndromes. Age is indicated in years (y), disease duration and time of clinical follow up in months (m). The clinical scores Hoehn and Yahr (H/Y) scale and the score of the motor part of the unified parkinson's disease rating scale (UPDRS III) under best medication/deep brain stimulation (ON) and after >12 h without dopaminergic medication or deep brain stimulation (OFF) are depicted.

### Routine Parameters RNFL and macular thickness

The peripapillar RNFL, paramacular thickness and volume, and the thickness of the different retinal layers were measured as illustrated in figure 1A, the results of all retinal parameters for the different groups are depicted in table 2. The mean peripapillar RNFL did not differ significantly between groups (figure 1B). The mean total macular thickness and volume only of PSP patients (302±5.0 µm; 0.95±0.02 mm^3^) presented a significant reduction compared to both controls (318.2±2.82 µm; 1.02±0.01 mm^3^) and PD patients (317.5±2.6 µm; 1.01+−0.01 mm^3^) while the reduction observed in MSA (306.6±4.2 µm; 0.98±0.01 mm^3^) and CBS (311.5±3.3 µm; 1.00±0.01 mm^3^) failed to reach significance (figure 1C, volume not shown). The reduction of the macular thickness in PSP affected both the peripheral and the central part of the retina (reduction of 4.85% and 5.4% respectively, table 2), and for the peripheral paramacular retinal thickness MSA patients also differed significantly from controls (reduction of 4.1%, table 2).

**Table 2:** Complete dataset of OCT-parameters.

	Control	Control	PD	PD	MSA	MSA	PSP	PSP	CBS	CBS
	Mean	SEM	Mean	SEM	Mean	SEM	Mean	SEM	Mean	SEM
Mean RNFL	99.13	1.587	97.33	1.61	93.79	1.92	93.8	2.84	94.17	3.32
Temporal RNFL	73.89	2.017	73.08	2.12	72.37	3.45	65.07	2.35	69.3	3.35
Nasal RNFL	71.99	2.341	73.49	2.40	68.18	2.23	75.4	4.58	72.5	3.84
Superior RNFL	121.6	2.782	117.2	2.70	115.9	3.7	116.5	4.77	123.2	4.87
Inferior RNFL	126.7	3.063	127.2	2.53	118.4	4.16	117.1	3.95	118.6	5.65
Mean total MT	317.6	2.691	317.4	2.66	308.2	4.13	302.3*	5.02	311.5	3.30
Mean central MT	340.9	2.91	341.6	2.71	332.3	4.52	322.5*	5.95	334.9	3.90
Mean peripheral MT	294.3	2.604	293.5	2.53	283.9	3.67	280*	4.43	289.3	2.92
MT sup. peripheral	296	2.706	296.3	2.43	284.6	3.80	280.3*	4.61	294.5	3.54
MT inferior peripheral	285.1	2.75	284.8	2.39	277.4	3.27	271*	4.44	280.9	2.71
MT temp. peripheral	283.6	2.383	283.8	2.29	273.7	3.53	273.6	5.24	276	2.46
MT nasal peripheral	312.1	2.941	310.1	2.58	299,4*	4.06	291.5*	3.86	306.5	4.19
MT superior central	343.1	2.737	343.6	2.85	334.7	5.00	326.6*	7.24	337	4.22
MT inferior central	340.7	3.119	342.3	2.90	330.5	4.26	320.8*	6.32	334.8	3.42
MT temporal central	333.8	2.989	334	2.68	327.2	4.93	318,5*	7.83	326.5	3.16
MT nasal central	346.1	3.166	346.6	2.77	336.8	4.50	324*	4.21	341.2	5.21
Mean total MV	1.01	0.01	1.01	0.01	0.99	0.01	0.95*	0.02	1.00	0.01
Mean central MV	0.53	0.01	0.54	0.01	0.52	0.01	0.51	0.01	0.53	0.01
Mean peripheral MV	1.49	0.01	1.49	0.02	1.44	0.02	1.37*	0.02	1.46	0.02
MV sup. peripheral	1.52	0.01	1.51	0.01	1,45*	0.02	1.35*	0.05	1.49	0.03
MV inferior peripheral	1.44	0.02	1.47	0.02	1.41	0.02	1.28*	0.04	1.42	0.02
MV temp. peripheral	1.43	0.02	1.42	0.02	1.39	0.02	1.34	0.04	1.39	0.02
MV nasal peripheral	1.58	0.02	1.55	0.02	1.54	0.03	1.47*	0.04	1.56	0.03
MV superior central	0.53	0.01	0.53	0.01	0.53	0.01	0,51*	0.01	0.53	0.01
MV inferior central	0.53	0.01	0.54	0.01	0.52	0.01	0.53	0.04	0.57	0.04
MV temporal central	0.52	0.01	0.53	0.01	0.51	0.01	0,50*	0.01	0.51	0.01
MV nasal central	0.54	0.01	0.54	0.01	0.53	0.01	0.51*	0.01	0.54	0.01
Mean RGC+IPL	98.7	1.60	99.79	1.28	96.08	1.946	87.25*	2.74	94.42	1.75
Nasal RGC+IPL	103.1	1.58	102.5	1.50	97.23	1.49	89.96*	2.86	97.11	1.87
Temporal RGC+IPL	94.26	1.73	97.06	1.25	94.93	2.79	84.54*	2.72	91.72	2.65
Mean INL	42.79	0.69	46.8*	0.93	42.08	1.57	39.38	0.96	43.64	3.15
Nasal INL	44.63	0.89	48.19	1.04	44.1	1.483	40.43	1.40	46.28	3.62
Temporal INL	40.96	0.65	45.4*	1.01	40.07	1.84	38.32	0.87	41	3.03
Mean OPL	34.59	1.03	33.74	0.73	34.38	1.36	38.21	1.66	38.36	2.71
Nasal OPL	36.14	1.59	35.21	0.84	34.53	1.56	43.5*	3.25	41.17	4.60
Temporal OPL	33.03	0.93	32.26	0.76	34.23	1.61	32.93	0.75	35.56	1.83
Mean central ONL	105.1	1.71	105.8	2.46	104	2.29	93.92*	2.17	116.3*	4.49

The means and the standard error of the means are indicated for all acquired parameters in the different groups. Abbreviations are as follows: RNFL = peripapillar retinal nerve fibre layer thickness in µm, MT = macular thickness in µm, MV = macular volume in mm3, RGC+IPL = retinal ganglion cell layer and inner plexiform layer measured together in µm, INL = inner nuclear layer in µm, OPL = outer plexiform layer in µm, ONL = outer nuclear layer in µm. Means that significantly differed from the control group are printed in bold and marked with an asterisk (*p*<0.05, ANOVA, Dunnett's multiple comparison post-hoc test).

### Manual segmentation

Due to the high resolution of the last generation spectral-domain OCT device used here, we were capable to identify the different retinal layers in transfoveal scans. We manually segmented the retinal layers in horizontal scans through the middle of the fovea (figure 2A) and measured the thickness of the different layers as indicated (figure 2B). The results are depicted in table 2.

The retinal ganglion cell- and inner plexiform layer (RGC+IPL) was reduced in PSP patients (87.3±2.7 µm) compared to controls (98.5±1.7 µm) and PD patients (99.8±1.3 µm) (figure 2C). Interestingly, the inner nuclear layer (INL) was thicker in patients with PD (46.8±0.9 µm) compared to both controls (42.8±0.8 µm) and patients with MSA (42.1±1.6 µm) and PSP (39.4±1.0 µm) (figure 2D). We observed no significant differences in thickness of the mean outer plexiform layer (OPL), however the nasal OPL was significantly thicker in PSP compared to controls (figure 2E, table 2). The thickness of the outer nuclear layer (ONL) was reduced in PSP (93.9±2.2 µm) compared to controls (105.9±1.7 µm), CBS (116.3±4.5 µm), and PD (107.8±3.6 µm) (figure 2F).

To evaluate the possible implications of our findings for clinical diagnostic procedures, we compared the intersections between the atypical parkinsonian syndromes and PD. The most pronounced differences were observed for ONL- and INL thickness between PSP and PD. ONL thickness alone differentiated our PSP from our PD patients with a sensitivity of 92% but with a sensitivity of only 51% using a cutoff of 104 µm thickness as positive for PSP.

### The ONL/OPL ratio and INL as indicators for PSP

As we observed reverse changes of ONL and OPL in our PSP patients, we considered calculating the ratio between the two layers to increase the discriminatory power. The ONL/OPL ratio of PSP patients differed indeed significantly from controls and patients with PD and CBS (figure 2G). Analyzing the INL/OPL ratio revealed a particular distribution in CBS patients clustering in two subgroups and a significant reduction in PSP compared to PD and controls (figure 2H). However, the ONL/OPL ratio showed the least overlap between these groups and thus served best for differentiation. Using the ONL/OPL with a cutoff of 3.1, we were able to differentiate between PSP and PD with a sensitivity of 96% and a specificity of 59%. As this parameter was not specific enough for our PSP patients, we used a combined test taking into account also the INL. Including the INL into the analysis with the constraint that only subjects with an INL below 46 µm were considered positive, we were able to raise the specificity to 70% with a sensitivity of 96% to differentiate PSP and PD in our cohort. The same test applied to the whole cohort differentiated between PSP and all the other Parkinson syndromes with a sensitivity of 96% and a specificity of 59%. As PSP is a rare disorder, we calculated the positive and negative likelihood ratios rather than the positive and negative predictive values to describe the value of the test. We observed a good negative likelihood ratio of 0.06 to discriminate between PSP and PD and 0.07 to discriminate against all other parkinsonian syndromes. Due to the rather low specificity of the test, the positive likelihood ratios for PSP were only 3.4 for discrimination against PD and 2.4 for discrimination against all Parkinson syndromes. No other combination of tests or ratios was better able to differentiate between PSP and PD or the other Parkinson syndromes.

### Correlations

It is noteworthy that we observed no correlation between the visual acuity of patients, the duration of disease, or the UPDRS-III with the mean peripapillar RNFL, macular thickness, macular volume, or any of the segmented retinal layers in any of the Parkinson syndromes (Spearman, data not shown). The OFF UPDRS-III of our PD patients did not differ from MSA, CBD, or PSP and the ON UPDRS-III did not differ between MSA, CBD and PSP indicating a similar severity of disease.

## Discussion

The differential diagnosis of Parkinson syndromes is still a clinical one and not always easy, especially at the onset of disease, when clinical symptoms are mild and the responsiveness to L-Dopa is ambiguous. Often, the final diagnosis can only be established taking into account the further course of disease. Several paraclinical tests including SPECT, MRI and transcranial sonography have been developed to facilitate the diagnostic process [38], [39], the present study is the first to systematically evaluate retinal changes in the differential diagnosis of Parkinson syndromes.

Our data indicate that different parkinsonian syndromes are associated with distinct retinal alterations. The INL is thicker in PD and thinner in PSP, while the ONL is thicker in CBS and thinner in PSP. Calculating the ratio between the ONL and OPL helped to identify patients with PSP. A combined test that also considers changes in the INL can differentiate PSP from PD and other Parkinson syndromes with a sensitivity of 96%. A good negative likelihood ratio (<0.1) for this test indicates that a negative result strongly argues against PSP. However, the specificity to discriminate against the other Parkinson syndromes was only 59% and against PD 70%. This translates to only fair positive likelihood ratios (<4) which indicates that a positive test result can be interpreted only as a hint favoring PSP and that the other differential diagnoses are not ruled out.

The diagnostic relevance of changes in macular thickness is not as easy to interpret. Our patients with MSA also had a non-significant tendency to thinner paramacular layers and the previously reported reduction of RNFL thickness in MSA failed to reach significance in our study [30]. However, our MSA patients presented a mean RNFL very close to the previous study (93.8+/−1.9 µm versus 91.9+/−1.45 µm) [30] and an isolated comparison of MSA patients and controls using a t-test showed significance also for our cohort. Some previous publications on PD [24], [25], [26] did report reductions of peripapillar RNFL or macular thickness while others [28], [29] did not, which was also the case in the presented work. As the clinical differentiation of PD from the atypical parkinsonian syndromes often necessitates taking in account the course of disease and the previous studies do not report neurological follow up, a possible explanation for the discrepancies between studies could be that a percentage of atypical patients were enrolled. We represent the study with the largest number of PD patients to date and present data on patients who were diagnosed at a specialized center for movement disorders and clinically followed up over time, which largely reduces the risk of diagnostic inaccuracy (supplemental table S1).

The functional consequences of the retinal changes observed here are unknown and studies including functional tests like low contrast letter recognition, multifocal ERGs and VEPs are already underway to address this point.

Morphometric MRI studies have demonstrated brain atrophy beyond the dopaminergic neurons of the substantia nigra not only in atypical parkinsonian syndromes but also in PD [2], [3], [4], [40], [41]. In the retina, dopaminergic cells are mainly amacrine cells located in the INL [42], [43], and several functional studies have demonstrated the importance of dopamine signaling for the visual system (reviewed in [44]). However, the previously reported reductions of RNFL and inner retina in PD [24], [25], [26] and reduction of RNFL in MSA [30] are due to changes affecting the RGCs or their axons. We observed changes of different retinal layers involving different cell types. The ONL, which contains the granule cells that link the rods and cones to the synaptic contacts in the OPL, was thinner in PSP. We observed no significant differences of the mean OPL where the synaptic contacts between cells from the ONL and INL take place, however the nasal OPL alone was thicker in PSP patients. Because in most patients the IPL could not be precisely distinguished from the RGC layer, we measured these layers together. The RGC+IPL contains the fibers of Mueller and the synaptic contacts from the IPL as well as the retinal ganglion cells, which make up the axons of the optic nerve. This layer was reduced in PSP patients and we hypothesize that in a larger collective the tendency to a thinner RGC+IPL observed in MSA would also be significant.

Our finding that PSP and MSA patients present the most profound retinal degeneration reflects what is seen in the brain of these patients in comparative MRI studies, where morphometry revealed the most pronounced atrophy in these two entities compared to PD and controls [45], [46], [47]. The molecular mechanisms underlying the retinal changes leave room for speculation and should be investigated in a large histopathological study. Possibly aggregation of protein tau might play a role in the retinal degeneration observed in PSP. Tau deposition has been detected in adult human retinae [48] and Alzheimer's disease, another common tauopathy, which also shows pronounced retinal degeneration [49], [50], [51], [52].

Our CBS cohort presented rather large variations in most retinal parameters and the macular thickness and the ratio of INL/OPL (figure 2H) even seemed to cluster in two subgroups. A recent clinicopathological study re-evaluating cases clinically diagnosed as corticobasal syndrome revealed a remarkably low positive predictive value of the clinical diagnosis of CBS for pathologically confirmation of corticobasal degeneration [53]. Possibly the variations observed in our cohort of patients with clinical CBS resemble different pathologies.

The data presented here suggest that especially patients with PSP may be interesting subjects for further functional studies of the visual system as they may give insight into the functional consequence of the morphological changes observed.

The fact that we observed no correlation between disease duration and UPDRS-III scores in our cohort of patients with manifest parkinsonian syndromes suggests that the retinal changes occur already early in disease and may not show a pronounced progression over time. Similar observations have been made for transcranial sonography, where hyperechogenicity is already observed early in PD (reviewed in [54]). This is of interest, as it suggests that OCT might play a future role in the early differential diagnosis of patients with Parkinson syndromes.

OCT is a fast, non-invasive, and cost-efficient method to assess retinal morphology. The next generation of OCT devices is likely to feature an automated segmentation of the different paramacular retinal layers. This will largely facilitate similar studies in parkinsonian syndromes and may open up the possibility to offer OCT as a diagnostic tool for patients with movement disorders. Our data suggest that the INL and ONL may be of special interest in parkinsonian syndromes, as they were reversely affected in PD, CBS, and PSP and thus may help to discriminate these diseases.

## Supporting Information

Table S1 **Key clinical features.** The key clinical features of all patients are depicted. Abbreviations are as follows: ON/OFF = score of the motor part of the unified parkinson's disease rating scale (UPDRS III) under best medication (and stimulation if applicable)/after >12 h without dopaminergic medication, F = female, M = male, y = years, m = months, AS = asymmetric manifestation, SY = symmetric manifestation, MF = motor fluctuations, DBS = deep brain stimulation, DUO = Duodopa pump treatment, APO = Apomorphin pump treatment, AC = antecollis, OD = orthostatic dysregulation, UI = urinary incontinence, PI = postural instability, BS = bulbar signs, AP = apraxia, Dy = dystonia, PLTDR = positive long term L-Dopa response, NLTDR = negative long term L-Dopa response.
(DOC)
