# Supplementary material for: Optical Coherence Tomography in Parkinsonian Syndromes
Source: PLoS One. 2012 Apr 13;7(4):e34891. doi: 10.1371/journal.pone.0034891 (PMC3325949; doi:10.1371/journal.pone.0034891)
Supplement: Table S1 — Key clinical features. The key clinical features of all patients are depicted. Abbreviations are as follows: ON/OFF = score of the motor part of the unified parkinson's disease rating scale (UPDRS III) under best medication (and stimulation if applicable)/after >12 h without dopaminergic medication, F = female, M = male, y = years, m = months, AS = asymmetric manifestation, SY = symmetric manifestation, MF = motor fluctuations, DBS = deep brain stimulation, DUO = Duodopa pump treatment, APO = Apomorphin pump treatment, AC = antecollis, OD = orthostatic dysregulation, UI = urinary incontinence, PI = postural instability, BS = bulbar signs, AP = apraxia, Dy = dystonia, PLTDR = positive long term L-Dopa response, NLTDR = negative long term L-Dopa response. (DOC) [file pone.0034891.s001.doc]

**Supplemental table S1: Key clinical features**

|  |  |  |  |  |  | UPDRS-III | |
| --- | --- | --- | --- | --- | --- | --- | --- |
| Age, y | Sex | Diagnosis | Duration, y | Follow up, m | Key clinical features | ON | OFF |
| 48 | M | PD | 21 | 22 | As, PLTDR, AC, MF, DBS | 6 | 52 |
| 72 | M | PD | 20 | 19 | AS, PLTDR, MF | 24 | 29 |
| 75 | M | PD | 16 | 120 | AS, PLTDR, MF, PI, DBS | 9 | 30 |
| 57 | M | PD | 15 | 30 | AS, PLTDR, MF, DBS | 15 | 54 |
| 67 | M | PD | 15 | 19 | AS ,PLTDR, MF | - | - |
| 45 | M | PD | 14 | 22 | AS, PLTDR, MF | 21 | 42 |
| 71 | M | PD | 14 | 16 | AS, PLTDR, MF, PI | 22 | - |
| 71 | M | PD | 13 | 21 | AS, PLTDR, MF, DUO | 21 | 34 |
| 73 | M | PD | 12 | 45 | AS, PLTDR, MF, PI, DBS | 10 | 27 |
| 72 | M | PD | 12 | 44 | AS, PLTDR, MF, DBS | 32 | 63 |
| 55 | M | PD | 11 | 51 | AS, PLTDR, MF, DUO | 24 | - |
| 69 | F | PD | 11 | 25 | AS, PLTDR, MF, DBS | 19 | 47 |
| 76 | M | PD | 11 | 21 | AS, PLTDR, MF | 22 | - |
| 65 | M | PD | 11 | 22 | AS, PLTDR, MF | 26 | 30 |
| 65 | M | PD | 10 | 56 | AS, PLTDR, MF, PI, BS, DBS | 25 | 48 |
| 64 | F | PD | 9 | 57 | AS, PLTDR, MF, PI, DBS | 28 | 62 |
| 52 | M | PD | 9 | 27 | AS, PLTDR, MF | 21 | 34 |
| 78 | M | PD | 9 | 26 | SY, PLTDR, PI | 41 | 45 |
| 73 | M | PD | 8 | 23 | AS, PLTDR, MF, DBS | 23 | 31 |
| 65 | F | PD | 8 | 21 | AS, PLTDR, MF, DBS | 11 | 42 |
| 61 | F | PD | 8 | 19 | AS, PLTDR, MF | 10 | 16 |
| 60 | M | PD | 7 | 28 | AS, PLTDR, MF | 19 | - |
| 81 | M | PD | 7 | 19 | AS, PLTDR | - | - |
| 44 | F | PD | 6 | 14 | AS, PLTDR | 18 | - |
| 73 | M | PD | 6 | 19 | AS, PLTDR | 23 | - |
| 49 | M | PD | 4 | 45 | AS, PLTDR, MF | 39 | - |
| 67 | M | PD | 3 | 53 | AS, PLTDR | 34 | - |
| 36 | M | PD | 3 | 42 | AS, PLTDR | 12 | 17 |
| 59 | M | PD | 3 | 22 | AS, PLTDR | 18 | 22 |
| 36 | M | PD | 2 | 27 | AS, PLTDR | 13 | 18 |
| 54 | F | PD | 2 | 27 | AS, PLTDR, MF, APO | 9 | 11 |
| 48 | F | PD | 2 | 19 | AS, PLTDR | 16 | - |
| 53 | F | PD | 4 | 48 | AS, PLTDR, MF | 24 | 43 |
| 85 | F | PD | 1 | 19 | AS, PLTDR | 22 | - |
| 55 | M | PD | 2 | 21 | AS, PLTDR | 12 | 13 |
| 67 | M | PD | 4 | 25 | AS, PLTDR, MF | 22 | - |
| 45 | M | PD | 2 | 32 | AS, PLTDR | 10 | 10 |
| 70 | M | PD | 2 | 26 | AS, PLTDR, MF, OD | 14 | 15 |
| 43 | M | PD | 2 | 19 | AS, PLTDR | 10 | 14 |
| 48 | F | PD | 3 | 19 | AS, PLTDR | 24 | - |
| 66 | M | MSA-P | 7 | 27 | SY, UI, BS, NLTR | 52 | 54 |
| 59 | M | MSA-P | 5 | 53 | SY, UI, BS, OD, NLTR | 41 | 44 |
| 72 | M | MSA-P | 4 | 37 | SY, UI, BS, OD, NLTR | 58 | 58 |
| 68 | M | MSA-P | 4 | 21 | SY, UI, NLTR | 25 | 28 |
| 63 | M | MSA-C | 3 | 38 | SY, UI, BS, NLTR | 32 | 31 |
| 74 | M | MSA-P | 1 | 25 | SY, UI, NLTR | 15 | 18 |
| 68 | M | MSA-P | 1 | 27 | SY, UI, NLTR | 38 | 42 |
| 65 | M | MSA-P | 10 | 6 | AS, UI, AC, NLTR | 27 | 31 |
| 54 | F | MSA-P | 13 | 57 | SY, UI, BS, NLTR | 54 | - |
| 66 | M | MSA-P | 2 | 12 | SY, UI, OD, BS, NLTR | 14 | 18 |
| 49 | M | MSA-P | 5 | 47 | AS, UI, BS, OD, NLTR | 26 | 26 |
| 68 | M | MSA-P | 4 | 15 | AS, UI, BS, OD, NLTR | 19 | 19 |
| 72 | M | MSA-P | 4 | 12 | SY, BS, NLTR | 26 | 26 |
| 59 | M | MSA-P | 5 | 20 | SY, UI, BS, NLTR | 25 | 23 |
| 62 | M | MSA-P | 5 | 6 | AS, UI, BS, AC, NLTR | 38 | 38 |
| 56 | M | MSA-C | 1 | 7 | SY, UI, BS, NLTR | 42 | - |
| 59 | F | MSA-P | 1 | 4 | AS, UI, BS, AC, NLTR | 38 | 42 |
| 60 | F | MSA-C | 5 | 5 | AS, UI, OD, NLTR | 48 | - |
| 50 | M | MSA-C | 2 | 22 | Sy, UI, BS, OD, NLTR | - | - |
| 72 | M | CBS | 4 | 40 | AS, Ap, Dy, UI, NLTR | 21 | 22 |
| 65 | M | CBS | 5 | 31 | AS, Ap, Dy, NLTR | 21 | 23 |
| 72 | F | CBS | 1 | 23 | AS, Ap, Dy, NLTR | 36 | 39 |
| 74 | M | CBS | 4 | 10 | AS, Ap, Dy, AC, NLTR | 46 | 48 |
| 56 | F | CBS | 1 | 7 | AS, AP, Dy, NLTR | 11 | - |
| 51 | F | CBS | 4 | 26 | AS,ALP, Ap, Dy, NLTR | 42 | 45 |
| 70 | F | CBS | 3 | 19 | AS, AP, NLTR | 16 | 20 |
| 57 | F | CBS | 2 | 8 | AS, AP, Dy, NLTR | 25 | - |
| 65 | F | CBS | 2 | 16 | AS, AP, NLTR | 9 | 9 |
| 56 | F | CBS | 1 | 4 | AS, Ap, NLTR | 8 | 8 |
| 63 | F | PSP | 3 | 36 | AS, SO, PI, BS, NLTR | 44 | - |
| 68 | F | PSP | 2 | 12 | SY, SO, PI, UI, NLTR | 13 | 16 |
| 80 | M | PSP | 2 | 9 | SY, SO, PI, NLTR | 23 | 23 |
| 73 | M | PSP | 5 | 34 | SY,SO, PI, NLTR | 26 | 28 |
| 75 | M | PSP | 8 | 25 | AS, SO, PI, NLTR | 39 | 36 |
| 77 | M | PSP | 10 | 11 | SY, SO, PI, NLTR | 44 | - |
| 71 | M | PSP | 12 | 31 | AS, SO, PI, BS, NLTR | 36 | 38 |
| 75 | M | PSP | 4 | 7 | SY, SO, PI, NLTR | 17 | 20 |
| 70 | M | PSP | 1 | 7 | SY, SO, PI, NLTR | 18 | 18 |
| 74 | M | PSP | 5 | 47 | SY, SO, PI, NLTR | 6 | 6 |
| 79 | F | PSP | 5 | 9 | AS, SO, PI, NLTR | 52 | 56 |
| 73 | M | PSP | 2 | 6 | SY, SO, PI, NLTR | 29 | 29 |
| 62 | M | PSP | 2 | 6 | SY, SO, BS, PI, UI, NLTR | 38 | - |
| 66 | M | PSP | 1 | 6 | SY, SO, PI, NLTR | 42 | 44 |
| 64 | M | PSP | 2 | 4 | AS, SO, PI, NLTR | 22 | - |

**Supplemental table S1: Key clinical features**

The key clinical features of all patients are depicted. Abbreviations are as follows: ON/OFF = score of the motor part of the unified parkinson’s disease rating scale (UPDRS III) under best medication (and stimulation if applicable) / after >12h without dopaminergic medication, F = female, M = male, y = years, m = months, AS = asymmetric manifestation, SY = symmetric manifestation, MF = motor fluctuations, DBS = deep brain stimulation, DUO = Duodopa pump treatment, APO = Apomorphin pump treatment, AC = antecollis, OD = orthostatic dysregulation, UI = urinary incontinence, PI = postural instability, BS = bulbar signs, AP = apraxia, Dy = dystonia, PLTDR = positive long term L-Dopa response, NLTDR = negative long term L-Dopa response.
